# The Book World of Medicine and Science

**Published:** 1899-09-30

**Authors:** 


### The Book World of Medicine and Science.

Asthma: Recent Advances in its Treatment. By Ernest Kingscote, M.B., C.M., &c. (London: Henry J. Glaisher. 1899. Pp. 168. Illustrated. Price 5s.)

Dr. Kingscote, who is well known to the profession as the author of several interesting papers on asthma and the treatment of heart disease is, on his own confession, an enthusiast. And in this book he has expanded his statement of his theories and has entered into a strong defence of them. In the first place Dr. Kingscote is persuaded of the value of an ingenious pleximeter of his own devising. This pleximeter, or rather pleximeter and plessor, is certainly a convenient and accurate mechanism for percussion, and in the hands of one accustomed to its use doubtless reveals much that is ordinarily undetected. But we are not sure that it is destined to supplant immediate percussion, and we recollect a friendly trial between Dr. Kingscote and a well-known physician, in which the latter, by palpation alone, detected quite as much as we fancy did Dr. Kingscote. But Dr. Kingscote's chief thesis is the importance of the treatment of cardiac dilatation in cases of asthma. No doubt Dr. Kingscote can bring forward some striking clinical records, and we have for long believed that practically the cardiac dilatation of asthmatics is much neglected. We are not prepared to deny that this form of cardiac dilatation is often suitably treated by gradual resistance exercises and by saline baths. We are more inclined to cavil at Dr. Kingscote's logic. For he deduces, as the result of his clinical work, the hypothesis that cardiac dilatation is very frequently the cause of asthma, as we understand him, by pressing on the vagus. Perhaps it were more logical to say that primary asthma very frequently brings about cardiac dilatation, often masked by emphysema; that the dilatation forms a link in a vicious circle, in which resulting pulmonary congestion aggravates the primary asthma; and that an early step in the treatment of asthmatics should be the removal of cardiac dilatation, and so of the secondary asthma, which is, so to speak, superimposed on the primary condition. Be that as it may, there can be no question of the practical beneficence of Dr. Kingscote's work, and there is much that is suggestive and stimulating in his theoretical disquisitions. Especially are the remarks on the real configuration of the heart *in situ* interesting and fresh. Dr. Kingscote makes no unwarranted claims, and puts his views forward with diffidence and a desire for discussion. We are quite sure that the profession will pay Dr. Kingscote the deserved compliment of careful consideration and of practical application.

Gleet and Chronic Diseases of the Urethra and Prostate. By G. Dalton, M.S.A.Lond. (London: H. Kempton. 1899. Pp. 48. Price 1s.)

This little book is not more than a digest of what every tolerably-educated practitioner is familiar with. Certainly Mr. Dalton is a little insistent on the merits of irrigation, of spring bougies, and of certain proprietary medicaments; and perhaps it is not more than a coincidence that Mr. Dalton's remarks are supplemented by the ample trade advertisements of these articles which follow the subject-matter proper of the book. We are bound to say, however, that Mr. Dalton has written his brochure pleasantly and succinctly, and displays sufficient practical acquaintance with his subject.

Constipation and its Modern Treatment. By George Herschell, M.D.Lond. (London: Henry J. Glaisher, 1899. Second edition. Pp. 90. Price 2s. 6d.)

We have so recently commented on Dr. Herschell's valuable and interesting essay that on the present occasion we need do little more than to express our pleasure at that public appreciation which has so soon resulted in the appearance of its second edition. As before, we cordially endorse Dr. Herschell's teaching, and commend it to the attention of every general practitioner. The book has been carefully revised and its value added to by a fairly complete index.

Manual for the Church Lads' Brigades Medical Corps. (Published at the Church House, Dean's Yard, S.W. Second edition. Pp. 122. 16mo. Price 6d.)

This little manual fulfils a purpose sufficiently indicated by the title. The matter is admirably and intelligently arranged, though necessarily derived in great part from the Manual of the Royal Army Medical Corps. It is bound to be of help and value to all concerned in the work of boys' cadet corps.

Burdett's Hospitals and Charities, 1899. Being the Year Book of Philanthropy and the Hospital Annual. By Sir Henry Burdett, K.C.B. (London: Scientific Press. 1899. Price 5s.)

It is rather late in the year for us to find ourselves reviewing an annual, and the author of this one, in his preface, not inappropriately expresses his regret at the unavoidable delay which has taken place in its production. This, he says, has been due to circumstances quite beyond his control, and he promises that in future "Burdett's Hospitals and Charities" shall appear punctually in March, which is well, for indeed those who have once got accustomed to depend upon this work could not comfortably do without it, so multifarious are the details entered into, and so accurate is the information given on the large number of subjects dealt with.

The directory portion of the book retains its old features, and in that part which is devoted to hospital finance all the usual details and tabulations on the subject will be found brought up to the date of the last reports available. Not the least important part of this, as of previous issues, lies in the preliminary chapters, in which the author gives his opinion on hospital affairs at large. As usual, he speaks somewhat *ex cathedrâ*. This at least has the advantage of giving these chapters a personal aspect, and thus renders them interesting, although perhaps some will dispute the conclusion arrived at. Sir Henry Burdett seems strongly convinced that lavish expenditure is becoming a "fashion" in all departments of a modern hospital; and against this he protests. "The more money which the public give to hospital purposes, the greater," he says, "seems to be the effort made to find excuses for the expenditure of larger and larger sums in every department." On the finances and general management of some hospitals he is very severe, and he says that "if in any way it were possible to place two sound men of business upon every hospital board—men who were impressed with the feeling that the present rate of expenditure is too high—the result would be that the cost per bed, instead of increasing, would be materially reduced, to the advantage of the institutions, and without affecting in any way the comfort of the inmates or the efficiency of the work done." Perhaps he is right. At any rate he is confident.

